# Hesperidin Attenuates Experimental MASH by Modulating the Liver–Immune–Brain Axis: Integrated Evidence from Network Pharmacology and In Vivo Analysis

**DOI:** 10.3390/nu18091402

**Published:** 2026-04-29

**Authors:** Seung-Hoon Yoo, Ji-Han Kim, Yeon-Joo Yoo, Byung-Cheol Lee

**Affiliations:** Department of Clinical Korean Medicine, Graduate School, Kyung Hee University, 26 Kyungheedae-ro, Dongdaemun-gu, Seoul 02447, Republic of Korea; ysh9306@khu.ac.kr (S.-H.Y.); ilpc21@khu.ac.kr (J.-H.K.); alice98113@khu.ac.kr (Y.-J.Y.)

**Keywords:** hesperidin, metabolic dysfunction-associated steatohepatitis, liver–immune–brain axis, neuroinflammation, oxidative stress, fibrosis, macrophages, network pharmacology

## Abstract

**Background/Objectives:** Metabolic dysfunction-associated steatohepatitis (MASH) is characterized by severe hepatic steatosis, lobular inflammation, and fibrosis. Although hesperidin, a citrus-derived flavanone, has been reported to exert metabolic and anti-inflammatory effects, its role in severe inflammatory and fibrotic conditions such as MASH remains incompletely understood. This study aimed to evaluate the effects of hesperidin in MASH using integrated in silico and in vivo approaches. **Methods:** Potential targets of hesperidin were identified using network pharmacology and molecular docking. For in vivo validation, C57BL/6 mice were fed a methionine- and choline-deficient (MCD) diet for five weeks, with oral administration of hesperidin (150 or 300 mg/kg/day) starting from week two. The MCD model induces severe hepatic inflammation and fibrosis but does not fully reflect metabolic features such as obesity and insulin resistance. Hepatic histology, serum transaminases, immune cell populations, and hypothalamic neuroinflammatory markers were assessed. **Results:** In silico analyses suggested that hesperidin interacts with key regulators associated with MASH, including PPARG, TGFB1, and TNF. In the in vivo MCD-induced model, hesperidin treatment reduced hepatic lipid accumulation and collagen deposition, accompanied by significant decreases in serum ALT and AST levels (by approximately 30–34% and 42–53%, respectively, depending on dose). These effects were associated with downregulation of pro-inflammatory and pro-fibrogenic gene expression and increased expression of antioxidant markers. In addition, hesperidin decreased circulating Ly6C^high^ monocytes and hepatic Kupffer cells, along with reduced hypothalamic microglial and astrocyte activation. **Conclusions:** Hesperidin attenuated key pathological features of MASH, including steatosis, inflammation, and fibrosis, and was associated with modulation of peripheral immune responses and central neuroinflammatory markers. These findings suggest that hesperidin may influence the liver–immune–brain axis and warrant further investigation in models that more closely reflect human metabolic conditions.

## 1. Introduction

Nonalcoholic fatty liver disease (NAFLD), recently redefined as metabolic dysfunction-associated steatotic liver disease (MASLD), represents a highly prevalent chronic liver disorder globally [1]. Its progressive inflammatory subtype, metabolic dysfunction-associated steatohepatitis (MASH), is histologically characterized by excessive hepatic steatosis, hepatocyte ballooning, lobular inflammation, and varying stages of fibrosis [2]. If left unmanaged, MASH can advance to severe clinical outcomes, including cirrhosis and hepatocellular carcinoma [3]. The pathogenesis of MASH is highly complex and is widely explained by the “multiple parallel hits” model [4], in which lipid accumulation sensitizes the liver to secondary insults such as mitochondrial dysfunction, oxidative stress driven by reactive oxygen species (ROS), and activation of innate immune responses, ultimately leading to hepatocellular injury and fibrogenesis [5].

To experimentally investigate the complex pathological features of MASH, the methionine- and choline-deficient (MCD) diet model has been widely employed [6]. The MCD diet model impairs hepatic lipid export by disrupting very-low-density lipoprotein (VLDL) synthesis and promotes mitochondrial defect [7]. Although this rodent model does not fully replicate the systemic metabolic syndrome features seen in human patients, such as obesity and insulin resistance [8], it reproducibly induces severe steatohepatitis, marked inflammatory cell infiltration, and early fibrosis within a short experimental period [9]. Therefore, the MCD model serves as a reliable and suitable platform for strictly evaluating the anti-inflammatory and anti-fibrotic potential of novel therapeutic candidates, although its limitations should be considered when interpreting translational relevance.

Currently, the clinical management of MASH remains a significant challenge due to the lack of widely approved and effective pharmacological therapies, highlighting an urgent need for alternative nutritional interventions and plant-derived bioactive compounds [10]. Citrus-derived flavanones are increasingly recognized for their diverse metabolic health benefits [11]. Hesperidin, a major bioactive flavanone abundantly present in *Citrus reticulata*, has been extensively studied for its potent antioxidant, anti-inflammatory, and lipid-lowering properties [12,13]. However, previous studies investigating the hepatoprotective effects of hesperidin have predominantly relied on high-fat diet (HFD) models [14,15], which often feature relatively mild inflammation and limited fibrosis [16]. Consequently, whether hesperidin exhibits substantial therapeutic effects in the severe, inflammation-driven, and fibrosis-prone environment of MCD-induced MASH remains largely unexplored [17].

Beyond hepatic pathology, MASH is increasingly recognized as a systemic disease involving multi-organ interactions and immune–neuro axis dysregulation [18]. Hepatic inflammation can promote the release of circulating cytokines and chemokines, which influence peripheral immune cell dynamics, including monocyte recruitment and macrophage activation [19,20]. In parallel, these systemic inflammatory signals may affect the central nervous system, particularly the hypothalamus, where microglial and astrocytic activation contribute to neuroinflammatory responses associated with metabolic dysregulation [21]. Despite these advances, the role of liver–immune–brain interactions in MASH progression remains incompletely understood, and whether bioactive compounds such as hesperidin can modulate this integrated axis has not been fully elucidated [22].

Therefore, we hypothesized that hesperidin exerts protective effects against MASH by modulating not only hepatic lipid metabolism and fibrogenesis but also systemic immune responses and central neuroinflammatory processes. To test this hypothesis, we investigated the effects of hesperidin in an MCD diet-induced MASH mouse model, integrating in vivo experimental validation with network pharmacology and molecular docking approaches to explore its multi-target mechanisms of action.

## 2. Materials and Methods

### 2.1. In Silico Network Pharmacology and Molecular Docking

To evaluate the pharmacokinetic characteristics of hesperidin, public databases including the Traditional Chinese Medicine Systems Pharmacology (TCMSP) Database, SwissADME, and PubChem were utilized to screen oral bioavailability (OB), drug-likeness (DL), and Caco-2 permeability. Although hesperidin did not fully meet the conventional ADME screening criteria (OB ≥ 30%, DL > 0.18, Caco-2 > −0.4), it was included in this study because it is a major bioactive compound abundantly present in *Citrus reticulata* and is metabolized in vivo into the active aglycone hesperetin, which exhibits higher bioavailability and potent pharmacological activity [23]. Potential molecular targets of hesperidin were predicted using the SwissTargetPrediction platform, while MASH-associated genes were retrieved from the GeneCards database. Overlapping genes between the two datasets were identified using the VENNY 2.1 tool and subsequently subjected to Gene Ontology (GO) and Kyoto Encyclopedia of Genes and Genomes (KEGG) pathway enrichment analyses via the DAVID bioinformatics resource. A protein–protein interaction (PPI) network was constructed using Cytoscape ver. 3.10.4. to identify core hub genes. Furthermore, molecular docking simulations were performed using PyRx ver. 1.2. (AutoDock Vina) to assess the binding affinities between hesperidin and representative MASH-related targets, and the resulting interactions were visualized using PyMOL ver. 3.1.

### 2.2. Chemicals and Preparation

Hesperidin (purity ≥ 98%) and pioglitazone was purchased from Tokyo Chemical Industry Co., Ltd. (Tokyo, Japan). The compound was dissolved in sterile normal saline, freshly prepared prior to administration, and stored at 4 °C in the dark. Pioglitazone (PIO), utilized as a positive control, was prepared accordingly.

### 2.3. Animal Experiments and Dietary Interventions

All animal procedures were conducted in accordance with institutional guidelines and approved by the Institutional Animal Care and Use Committee (IACUC) of Kyung Hee University. Six-week-old male C57BL/6 mice were obtained from Central Lab Animal, Inc. (Seoul, Republic of Korea) and housed under specific pathogen-free conditions (22 ± 2 °C, 40–70% humidity, 12 h light/dark cycle) with ad libitum access to food and water. To ensure high standards of animal welfare, the mice were clinically monitored on a daily basis by both the research team and the animal facility technicians. Following a one-week acclimation period, the mice were randomly divided into five groups (*n* = 5) based on previous studies [24]: Normal Chow (NC), methionine- and choline-deficient diet (MCD) (Dooyeol Biotech, Seoul, Republic of Korea), MCD + hesperidin 150 mg/kg/day (HES 150), MCD + hesperidin 300 mg/kg/day (HES 300), and MCD + pioglitazone 30 mg/kg/day (PIO) using a computer-generated random number sequence.

The dosages for hesperidin (150 and 300 mg/kg) were determined based on previous studies demonstrating its efficacy in mouse models of metabolic disorders and hepatic steatosis [25]. The dose of 30 mg/kg for PIO was selected as a positive control based on its established therapeutic effect in MASH animal models [26].

To induce MASH, the MCD diet was provided for four consecutive weeks. Beginning on the eighth day, hesperidin and PIO were administered daily via oral gavage for four weeks, while the NC and MCD groups received an equivalent volume of normal saline. Body weights and food intakes were monitored regularly throughout the experimental period. To monitor dietary consumption, food intake was measured regularly throughout the experimental period by weighing the remaining food in each cage. At the end of the experiment, the mice were anesthetized and euthanized via CO_2_ inhalation to harvest blood, liver, and epididymal fat pads.

### 2.4. Serum Biochemical Analysis

Blood samples collected via cardiac puncture were allowed to clot at room temperature and centrifuged at 3000 rpm for 20 min to separate the serum. The serum samples were stored at −40 °C until analysis. Serum levels of aspartate aminotransferase (AST) and alanine aminotransferase (ALT) were measured to assess hepatic injury. Additionally, comprehensive lipid profile parameters, including total cholesterol (TC), high-density lipoprotein (HDL), low-density lipoprotein (LDL), triglycerides (TG), non-esterified fatty acids (NEFA), and phospholipids (PL), were determined. Biochemical parameters were measured using commercially available ELISA kit (MyBioSource, San Diego, CA, USA) according to the manufacturers’ instructions. All assays were performed in duplicate by researchers who were unaware of the experimental group assignments, and absorbance was measured using a microplate reader under standardized conditions.

### 2.5. Histopathological and Immunohistochemical Analyses

Liver and brain tissues were fixed in 10% neutral buffered formalin, dehydrated through a graded ethanol series, and embedded in paraffin. Liver sections (4 µm) were stained with hematoxylin and eosin (H&E) to evaluate hepatic steatosis, and with Sirius Red to assess collagen deposition and fibrosis. The stained sections were observed under an light microscope (Olympus, Tokyo, Japan), and the positive areas were quantitatively analyzed using ImageJ software ver. 1.51. For immunohistochemical analysis of the brain, 3–4 µm sections were subjected to antigen retrieval using citrate buffer, followed by quenching of endogenous peroxidase with 3% H_2_O_2_ and blocking. The sections were then incubated with primary antibodies against IBA-1 and GFAP to assess glial activation in the hypothalamus, which were visualized using DAB or AEC chromogens. All histopathological analysis was conducted using a blinded method.

### 2.6. Quantitative Real-Time PCR (qRT-PCR)

Total RNA was isolated from frozen liver tissues using the Mini RNA Isolation II™ kit (Zymo Research, Irvine, CA, USA). The extracted RNA (1 µg) was reverse transcribed into complementary DNA (cDNA), and qRT-PCR amplification was performed using the 7900HT Fast Real-Time PCR System (Applied Biosystems, Carlsbad, CA, USA). The relative expression levels of genes involved in lipogenesis, inflammation, fibrosis, and oxidative stress were calculated using the 2^−ΔΔCt^ method, with GAPDH serving as the internal reference control.

### 2.7. Flow Cytometric Analysis (FACS)

Peripheral blood samples were collected in EDTA-coated tubes, and red blood cells were lysed prior to analysis. Following the blockade of non-specific binding with an Fc receptor blocking reagent, the cells were stained with fluorochrome-conjugated monoclonal antibodies targeting Ly6C and CD11b. After exclusion of debris and doublets based on forward and side scatter properties, leukocytes were gated, and monocyte populations were identified within the CD11b^+^ fraction. Ly6C^high^ and Ly6C^low^ subsets were defined according to fluorescence intensity. Unstained and single-stained controls were used to establish gating boundaries and perform compensation.

To analyze hepatic immune cell populations, liver tissues underwent enzymatic digestion using a cocktail of collagenase and DNase I. The resulting single-cell suspensions were filtered and subjected to a staining protocol consistent with the above-mentioned procedures. For flow cytometric identification, cells were labeled with specific antibodies, including CD45-APC Cyanine7, F4/80-APC, and CD11b-PE Cyanine7. All antibodies were purchased from BioLegend (San Diego, CA, USA). Kupffer cell subsets were defined by the expression of CD45^+^F4/80^+^ and CD45^+^F4/80^+^CD11b^+^ based on standard gating protocols. Flow cytometric analysis was performed using a FACS Canto flow cytometer (BD Biosciences, San Jose, CA, USA), and data were analyzed with FlowJo software ver. 10 (Tree Star Inc., Ashland, OR, USA).

### 2.8. Statistical Analysis

All data are expressed as the mean ± standard error of the mean (SEM). Statistical differences between experimental groups were analyzed using a one-way analysis of variance (ANOVA) followed by Tukey’s multiple comparison post hoc test via GraphPad Prism software (version 5.0; GraphPad Software, San Diego, CA, USA). A *p*-value of <0.05 was considered to indicate statistical significance.

## 3. Results

### 3.1. In Silico Pharmacokinetic Characterization of Hesperidin

To evaluate the drug-likeness and pharmacokinetic properties of hesperidin, an in silico screening was performed using the TCMSP database and SwissADME platform. Against the established criteria—oral bioavailability (OB) ≥ 30%, drug-likeness (DL) > 0.18, and Caco-2 permeability > –0.4—in silico ADME analysis indicated low oral bioavailability (13.33%) and low Caco-2 permeability (−2.03) for hesperidin. However, its drug-likeness well exceeded the commonly used threshold (DL = 0.67) (Table 1). Given this remarkably high drug-likeness score and its widely recognized in vivo pharmacological activities, hesperidin was ultimately identified as a highly viable and biologically active compound derived from *Citrus reticulata*, making it a suitable candidate for further investigation.

### 3.2. Network-Based Identification of Hesperidin Targets in MASH

**Figure 1:**
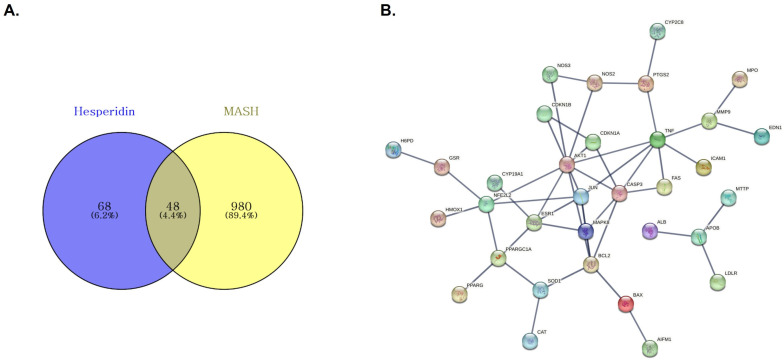
Identification and protein–protein interaction (PPI) network of the overlapping target genes between hesperidin and metabolic dysfunction-associated steatohepatitis (MASH). (**A**) Venn diagram illustrating the intersection of hesperidin-targeted genes and MASH-related genes. (**B**) PPI network of the 48 overlapping genes constructed using the STRING databases.

To elucidate the potential pharmacological targets of hesperidin against metabolic dysfunction-associated steatohepatitis (MASH), a network pharmacology approach was employed. A total of 116 hesperidin-associated target genes and 1028 MASH-related genes were retrieved from public databases. The intersection of these datasets yielded 48 overlapping genes, which were considered the core potential molecular targets of hesperidin in the context of MASH pathophysiology (Figure 1).

### 3.3. Functional Pathway and Gene Ontology Enrichment Analyses

**Figure 2:**
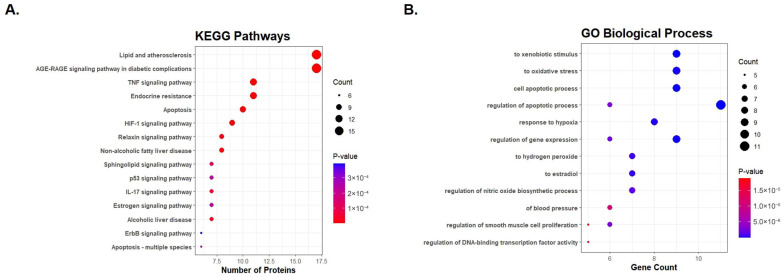
Key functional pathways and Gene Ontology (GO) enrichment analyses. (**A**) The most significantly enriched Kyoto Encyclopedia of Genes and Genomes (KEGG) pathways derived from the overlapping targets between hesperidin and MASH. (**B**) Representative bar charts summarizing the Gene Ontology (GO) enrichment analysis.

To systematically understand the biological roles of the 48 overlapping genes, KEGG pathway and Gene Ontology (GO) enrichment analyses were conducted. The KEGG analysis revealed that these targets were significantly enriched in pathways central to MASH progression, including lipid and atherosclerosis, AGE-RAGE signaling, TNF signaling, apoptosis, and oxidative stress. Furthermore, GO enrichment indicated that these targets are predominantly localized in the cytosol and intracellular organelles, executing critical molecular functions such as protein binding and enzyme regulation involved in inflammatory and fibrotic responses (Figure 2).

### 3.4. Molecular Docking Analysis of Hesperidin with Key MASH-Related Proteins

**Figure 3:**
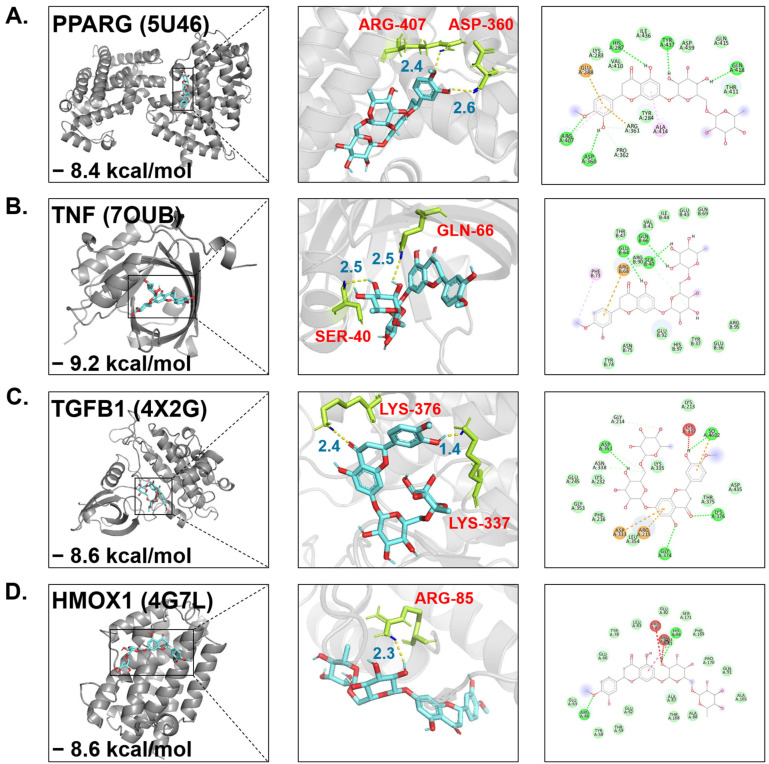
Representative molecular docking simulations of hesperidin with key MASH-related target proteins. Three-dimensional docking models illustrating the predicted binding poses and interaction sites of hesperidin with core regulatory proteins involved in (**A**) lipid metabolism, (**B**) inflammation, (**C**) fibrosis, and (**D**) oxidative stress.

To validate direct molecular interactions, molecular docking simulations were performed. Hesperidin exhibited strong binding affinities (binding energy ≤ −7.0 kcal/mol) with representative MASH-pathogenic proteins (Table 2). Specifically, structural simulations demonstrated that hesperidin forms highly stable complexes with targets centrally regulating lipogenesis and lipid metabolism (Figure 3A), as well as those driving inflammatory cascades, notably including TNF and IL-6 (Figure 3B). Furthermore, hesperidin exhibited strong binding capacities to key mediators of fibrogenesis such as TGFB1 (Figure 3C), and oxidative stress regulation (Figure 3D). These findings indicate that hesperidin can directly interact with core mediators across multiple pathogenic axes, providing a structural rationale for its in vivo therapeutic efficacy.

### 3.5. Effect of Hesperidin on Body Weight and Caloric Intake in MCD-Fed Mice

To investigate systemic metabolic alterations, body weight and daily caloric intake were monitored. Over the experimental period, the MCD diet induced a severe reduction in body weight compared to the normal chow (NC) group. Administration of hesperidin did not prevent this MCD-induced weight loss. Interestingly, despite the weight loss, the MCD group exhibited increased food intake, displaying a significantly higher daily food and caloric intake than the NC group. This elevated food consumption was further enhanced in the high-dose hesperidin (HES 300) group (Table 3).

### 3.6. Effect of Hesperidin on Liver and Epididymal Adipose Tissue Mass

Despite the overall body weight loss, hesperidin treatment significantly preserved specific organ masses. The profound reduction in epididymal fat pad mass and its ratio to body weight observed in the MCD group was significantly and partially reversed by hesperidin treatment. Concurrently, the MCD-induced reduction in absolute liver mass was significantly restored in the hesperidin-treated groups, which also led to a marked increase in the liver-to-body weight ratio (Table 3). These results indicated that hesperidin administration ameliorated the MCD diet-induced loss of liver and adipose tissue mass.

### 3.7. Effect of Hesperidin on Hepatic Histopathological Changes

**Figure 4:**
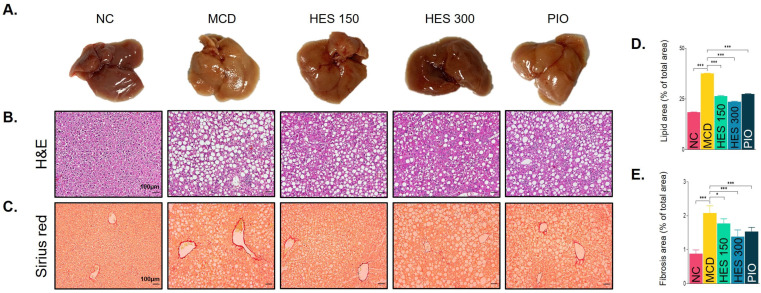
Hesperidin ameliorates macroscopic and histological hepatic steatosis and fibrosis in MCD-induced MASH mice. (**A**) Representative macroscopic images of the liver. (**B**) Representative histological sections stained with hematoxylin and eosin (H&E) (Scale bar = 100 μm). (**C**) Representative sections stained with Sirius Red (Scale bar = 100 μm). (**D**) Quantitative analysis of the hepatic lipid droplet area. (**E**) Quantitative analysis of the hepatic fibrosis area. Data are expressed as the mean ± SEM (*n* = 5–10 per group). * *p* < 0.05, *** *p* < 0.001 vs. MCD group.

The protective effect of hesperidin against hepatic injury was histologically confirmed. H&E staining revealed that the MCD diet induced massive hepatic lipid accumulation and cellular ballooning. However, hesperidin administration significantly attenuated the lipid droplet area. Furthermore, Sirius Red staining demonstrated prominent collagen deposition in the MCD group, whereas treatment with hesperidin significantly diminished the hepatic fibrotic area, demonstrating notable anti-fibrotic efficacy (Figure 4A–E).

### 3.8. Effect of Hesperidin on Serum Hepatic and Renal Biomarkers

**Figure 5:**
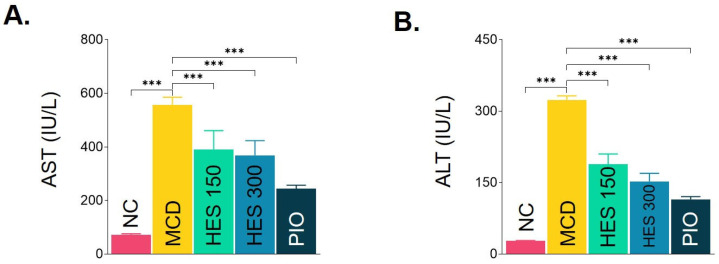
Effects of hesperidin on serum hepatic injury biomarkers. Bar graphs representing serum levels of (**A**) aspartate aminotransferase (AST), and (**B**) alanine aminotransferase (ALT). Comprehensive serum lipid profile data across all experimental groups are detailed in Table 4. Data are expressed as the mean ± SEM (*n* = 5–10 per group). *** *p* < 0.001 vs. MCD group. NC, normal chow; MCD, methionine- and choline-deficient diet; HES 150/300, MCD + hesperidin 150 or 300 mg/kg/day; PIO, MCD + pioglitazone 30 mg/kg/day.

To further evaluate the systemic effects of hesperidin, comprehensive serum biochemical analyses, including hepatic injury markers and lipid profiles, were performed (Table 4). To assess hepatocellular injury, serum transaminase levels were measured (Figure 5). The MCD diet provoked a dramatic elevation in both AST and ALT levels compared to the NC group. Conversely, hesperidin treatment significantly ameliorated these transaminase elevations, reflecting a substantial improvement in liver function (Figure 5A,B). Meanwhile, serum creatinine, which was elevated in the MCD group, did not exhibit statistically significant alterations following hesperidin treatment (Table 4).

### 3.9. Effect of Hesperidin on the Serum Lipid Profile

The MCD diet induced a distinctive, disease-specific reduction in circulating lipid parameters, including total cholesterol (TC), triglycerides (TG), HDL-C, LDL-C, phospholipids (PL), and free fatty acids (FFA). While hesperidin administration did not significantly alter most of these depleted lipid markers, the high-dose treatment (HES 300) resulted in a partial but significant recovery in serum TC levels compared with the MCD-alone group (Table 4).

### 3.10. Effect of Hesperidin on Circulating Monocytes and Hepatic Kupffer Cells

**Figure 6:**
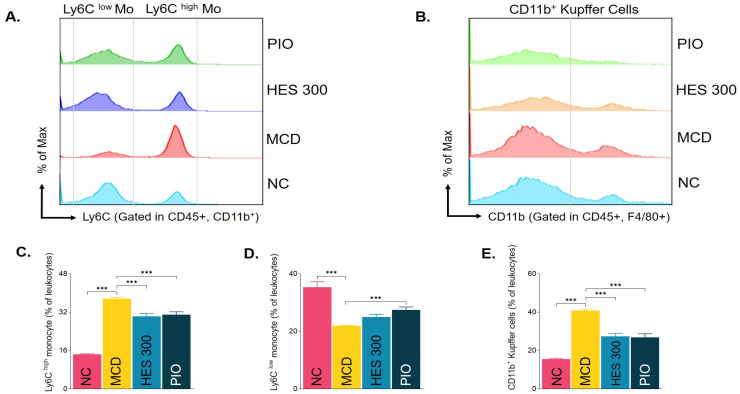
Effects of hesperidin on circulating pro-inflammatory monocytes and hepatic Kupffer cell populations. (**A**,**B**) Representative flow cytometric histograms of Ly6C and CD11b expressions. (**C**,**D**) Quantitative analysis of Ly6C^high^ and Ly6C^low^ monocyte percentages in leukocytes. (**E**) Quantitative analysis of CD11b^+^ Kupffer cells. Data are expressed as the mean ± SEM (*n* = 5–10 per group). *** *p* < 0.001 vs. MCD group.

Flow cytometric analysis demonstrated that the MCD diet substantially elevated the proportion of circulating pro-inflammatory Ly6C^high^ monocytes. Hesperidin treatment effectively reversed this systemic inflammatory state, significantly reducing the Ly6C^high^ subset. In parallel, the marked expansion of hepatic CD11b^+^ Kupffer cells observed in the MCD group was significantly suppressed by hesperidin, indicating an effective attenuation of macrophage-mediated inflammatory cascades in the liver (Figure 6A–E).

### 3.11. Effect of Hesperidin on Hepatic Metabolic, Inflammatory, and Fibrotic Gene Expressions

**Figure 7:**
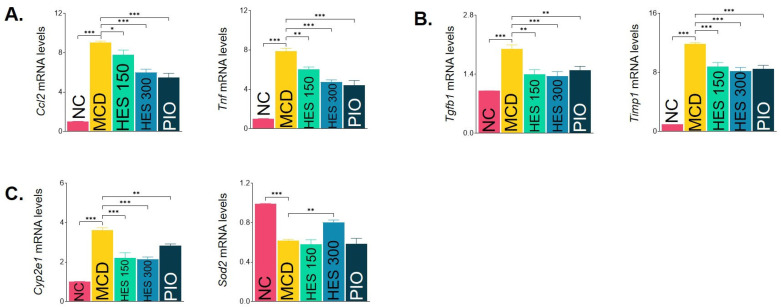
Key regulatory patterns of hepatic mRNA expressions and heatmap visualization. Relative hepatic mRNA expression levels of selected key genes involved in (**A**) inflammation (*Ccl2*, *Tnf*), (**B**) fibrosis (*Tgfb1*, *Timp1*), and (**C**) oxidative stress (*Cyp2e1*, *Sod2*) determined by quantitative real-time PCR. Comprehensive relative quantification values for all evaluated genes are detailed in Table 5. Data are expressed as the mean ± SEM (*n* = 5–10 per group). * *p* < 0.05, ** *p* < 0.01, *** *p* < 0.001 vs. MCD group. NC, normal chow; MCD, methionine- and choline-deficient diet; HES 150/300, MCD + hesperidin 150 or 300 mg/kg/day; PIO, MCD + pioglitazone 30 mg/kg/day. *Ccl2*, C-C motif chemokine ligand 2; *Tnf*, tumor necrosis factor; *Tgfb1*, transforming growth factor beta 1; *Timp1*, TIMP metallopeptidase inhibitor 1; *Cyp2e1*, cytochrome P450 family 2 subfamily E member 1; *Sod2*, superoxide dismutase 2.

To comprehensively elucidate the underlying molecular mechanisms, quantitative alterations in hepatic transcriptional programs related to lipid metabolism, inflammation, fibrosis, and oxidative stress were evaluated (Table 5). As visually summarized by the representative key markers (*Ccl2*, *Tnf*, *Tgfb1*, *Timp1*, *Cyp2e1*, *Sod2*) and heatmap analysis in Figure 7, the MCD diet severely dysregulated these metabolic and inflammatory gene expressions. Following hesperidin treatment, the MCD-induced overexpression of key inflammatory mediators, including *Adgre1*, *Ccl2*, and *Tnf*, was profoundly suppressed (Figure 7A, Table 5). Furthermore, the elevated expression of fibrogenic genes such as *Col3a1*, *Tgfb1* and *Timp1* was significantly downregulated (Figure 7B, Table 5). In addition to mitigating inflammation and fibrosis, hesperidin normalized oxidative stress-related markers by downregulating *Cyp2e1* and upregulating the endogenous antioxidant enzyme *Sod2* (Figure 7C, Table 5). Comprehensive profiling also revealed that hesperidin partially restored the expression of lipid metabolism-associated genes (*Srebf1* and *Ppara*), underscoring its multi-target regulatory efficacy across the MASH pathogenic cascade.

### 3.12. Effect of Hesperidin on Hypothalamic Glial Cell Activation

**Figure 8:**
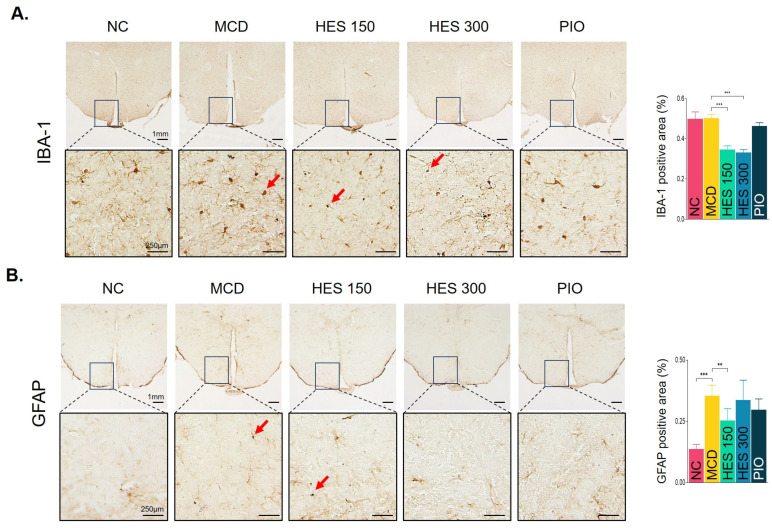
Hesperidin mitigates hypothalamic microglial and astrocyte activation in the liver-brain axis. Representative immunohistochemical images and quantitative analyses of (**A**) IBA-1 (microglia) and (**B**) GFAP (astrocytes) in the hypothalamus. Red arrows indicate activated glial cells (scale bars = 1 mm, 500 μm, and 250 μm). Data are expressed as the mean ± SEM (*n* = 5 per group). ** *p* < 0.01, *** *p* < 0.001 vs. MCD group. NC, normal chow; MCD, methionine- and choline-deficient diet; HES 150/300, MCD + hesperidin 150 or 300 mg/kg/day; PIO, MCD + pioglitazone 30 mg/kg/day. IBA-1, ionized calcium-binding adaptor molecule 1; GFAP, glial fibrillary acidic protein.

To explore the central anti-inflammatory effects of hesperidin, neuro-glial activation in the hypothalamus was evaluated. The MCD diet triggered significant astrocyte activation, evidenced by elevated GFAP expression, which was significantly mitigated by hesperidin treatment. Furthermore, hesperidin profoundly suppressed microglial IBA-1 expression compared to the MCD group. These findings suggest that the therapeutic actions of hesperidin extend to the central nervous system, modulating the liver–immune–brain axis (Figure 8A–B).

## 4. Discussion

In the present study, we demonstrate that hesperidin exhibits integrated protective effects against MCD diet-induced MASH by modulating key pathological processes, including hepatic lipid accumulation, inflammation, oxidative stress, and fibrosis, as supported by improvements in serum biochemical parameters such as ALT and AST levels. Importantly, these findings extend beyond simple phenotypic improvements and suggest that hesperidin modulates interconnected pathways underlying MASH progression at both peripheral and central levels.

Although the MCD diet model does not fully replicate the metabolic features of human MASH [31], such as obesity and insulin resistance, it remains a well-established model for inducing severe hepatic inflammation and rapid fibrogenesis [32]. In this context, our findings provide meaningful evidence that hesperidin retains its therapeutic potential even under highly inflammatory and fibrotic conditions. While previous studies have predominantly utilized high-fat diet models-characterized by relatively mild inflammation [33]—our study demonstrates that hesperidin effectively mitigates hepatic injury and lipid accumulation even in the relatively severe pathological environment of MCD-induced MASH. These results suggest a broader therapeutic applicability of hesperidin across diverse stages of metabolic liver disease.

MASH progression is closely associated with the activation of the innate immune system, particularly the recruitment and activation of the monocyte–macrophage axis [34]. In line with previous reports highlighting the anti-inflammatory properties of hesperidin [35], our results further demonstrate its capacity to regulate systemic immune responses. Specifically, hesperidin significantly reduced the proportion of circulating pro-inflammatory Ly6C^high^ monocytes and suppressed hepatic CD11b^+^ Kupffer cells [36], indicating suppression of both peripheral immune activation and intrahepatic inflammation. These cellular changes were supported by the downregulation of key pro-inflammatory genes, including *Adgre1*, *Ccl2*, and *Tnf*. Taken together, these findings suggest that hesperidin not only attenuates hepatic inflammation but also modulates systemic immune dynamics that contribute to disease progression [37].

Chronic inflammation and oxidative stress are major drivers of hepatic fibrogenesis [38]. Our results showed that hesperidin markedly reduced fibrotic area, as evidenced by Sirius Red staining, and suppressed the expression of key fibrogenic genes, including *Tgfb1*, *Col3a1*, and *Timp1*. These effects are likely mediated through both anti-inflammatory and antioxidant mechanisms. Notably, hesperidin inhibited the overexpression of *Cyp2e1*, a major source of reactive oxygen species (ROS), while restoring antioxidant enzymes such as *Sod2* and *Cat* [39]. These findings are consistent with previous studies demonstrating the antioxidative properties of hesperidin [40], but further extend its role to the regulation of fibrosis-related pathways in a severe MASH model [41].

Importantly, our study highlights the potential involvement of the liver–immune–brain axis in the therapeutic effects of hesperidin. Emerging evidence suggests that MASH is not a liver-restricted disease but involves systemic neuroimmune interactions [22]. In the present study, the MCD diet induced significant activation of hypothalamic astrocytes (GFAP) and microglia (IBA-1) [42], which was markedly attenuated by hesperidin treatment. These findings suggest that peripheral hepatic inflammation may influence central nervous system responses through immune-mediated signaling pathways [43]. This reciprocal crosstalk between the liver and brain has been increasingly recognized as a critical component of metabolic disease progression [44]. In this context, hesperidin may exert its therapeutic effects by interrupting this pathological crosstalk, thereby providing a novel mechanistic perspective beyond conventional hepatocentric approaches [45].

Despite these promising findings, several limitations should be considered. First, although network pharmacology and molecular docking analyses predicted multiple potential targets, some inconsistency was observed between in silico predictions and in vivo gene expression for certain targets, which may be attributed to the complex pharmacokinetics of hesperidin or model-specific factors [46]. Second, the MCD diet model lacks key metabolic features such as obesity and insulin resistance [47], which limits the direct clinical translatability of the findings. Therefore, further validation in alternative models, such as high-fat diet-induced MASH, is needed.

Another important consideration is the relatively low oral bioavailability of hesperidin. Once ingested, hesperidin is metabolized by intestinal microbiota into its aglycone form, hesperetin, which exhibits higher absorption and biological activity [48]. Therefore, the observed in vivo effects may be partially mediated by hesperetin rather than hesperidin itself [49]. This highlights the need for future studies to directly compare the efficacy of hesperidin and hesperetin, as well as to explore strategies to enhance bioavailability, including advanced delivery systems.

Additionally, while our findings support the involvement of the liver–immune–brain axis in the therapeutic effects of hesperidin, the precise mechanisms underlying this inter-organ crosstalk remain to be fully elucidated, and further studies are required to explore the liver-brain axis in greater depth as an integrated molecular pathway. Mechanistically, this axis may be mediated by circulating inflammatory cytokines and immune cell signaling [50], which can influence hypothalamic glial activation and central metabolic regulation. In particular, the reduction in pro-inflammatory monocytes observed in this study may contribute to decreased neuroinflammatory signaling, providing a potential link between peripheral immune modulation and central nervous system responses. However, direct causality between hepatic inflammation and hypothalamic neuroinflammation were not established in this study, warranting further mechanistic investigations.

Overall, our integrated in silico and in vivo analyses demonstrate that hesperidin exerts multi-target protective effects against MCD-induced MASH. By simultaneously attenuating hepatic steatosis, suppressing systemic and hepatic inflammation, reducing fibrogenesis, and modulating hypothalamic glial activation, hesperidin acts as a potential regulator of the liver–immune–brain axis. These findings provide compelling evidence for considering hesperidin as a promising therapeutic candidate for metabolic steatohepatitis and related systemic metabolic disorders.

## 5. Conclusions

In conclusion, the present study highlights that hesperidin exerts multi-target protective effects against MCD diet-induced metabolic dysfunction-associated steatohepatitis (MASH). Hesperidin administration significantly alleviated hepatic lipid accumulation, ameliorated hepatocellular injury markers, and profoundly suppressed the expression of key genes associated with inflammation and fibrogenesis. Furthermore, hesperidin normalized systemic immune profiles by reducing the proportion of circulating pro-inflammatory Ly6C^high^ monocytes and suppressing hepatic Kupffer cell expansion. Notably, it also attenuated microglial activation in the hypothalamus, highlighting its integrated modulatory effects on the central and peripheral inflammatory cascades. These in vivo outcomes strongly support our initial in silico predictions, which identified hesperidin as a potent modulator of core metabolic and inflammatory pathways. Therefore, these findings suggest that hesperidin holds great promise as a novel and safe therapeutic candidate for the management of MASH and its systemic complications via the regulation of the liver–immune–brain axis. In particular, the suppression of hepatic inflammatory signaling and fibrogenic mediators, together with the reduction of circulating Ly6C^high^ monocytes and hepatic Kupffer cells, may limit systemic inflammatory signaling, thereby contributing to the attenuation of hypothalamic microglial and astrocytic activation observed in this study. Further preclinical and clinical studies focusing on its detailed pharmacokinetic profiles, optimal dosing parameters, and long-term safety will be necessary to facilitate its translational application.

## 6. Limitations of the Study

Several limitations should be acknowledged when interpreting the present findings. First, although the methionine- and choline-deficient (MCD) diet is an established model for inducing steatohepatitis and fibrosis, it does not adequately reproduce key metabolic features of human MASH, particularly obesity-driven insulin resistance, which may restrict the translational applicability of the results.

Second, the limited number of animals included in each group may affect the reliability of statistical analyses and reduce the extent to which these findings can be generalized. Third, the target identification strategy based on network pharmacology and molecular docking primarily provides in silico estimations, and these proposed interactions were not experimentally confirmed at the level of protein expression or intracellular signaling cascades.

Fourth, central inflammatory changes were evaluated using a narrow set of glial activation markers, and a broader characterization of neuroinflammatory mediators, as well as functional assessments such as behavioral or cognitive outcomes, was not performed. In addition, the pharmacokinetic profile of hesperidin was not directly examined, including its low bioavailability, absorption, metabolic transformation into bioactive compounds such as hesperetin, and tissue distribution, which may influence its in vivo efficacy.

Finally, due to the relatively short duration of the study and its endpoint-based design, it was not possible to determine long-term therapeutic effects or to fully characterize dose-dependent responses. Further investigations addressing these aspects will be necessary to strengthen the interpretation of the current results.

## 7. Future Perspectives

Further research is required to extend the current findings and to better define the therapeutic relevance of hesperidin in metabolic dysfunction-associated steatohepatitis (MASH). In particular, validation in experimental systems that more closely reflect human metabolic conditions, such as diet-induced obesity or metabolically driven MASH models, will be important to improve translational relevance.

In addition, future investigations should focus on mechanistic confirmation of the potential molecular targets suggested by in silico analyses, including detailed characterization of protein-level changes, downstream signaling events, and functional outcomes.

A more comprehensive exploration of the liver–immune–brain axis is also warranted, incorporating broader profiling of central inflammatory mediators together with functional readouts such as behavioral and cognitive assessments, which may provide deeper insight into systemic regulatory networks.

Moreover, dedicated pharmacokinetic and metabolic studies are needed to better understand the absorption, biotransformation, and distribution of hesperidin and its active metabolites, including hesperetin, which are likely to contribute to its biological activity.

Finally, clinical validation through well-designed human studies, including randomized controlled trials, will be essential to establish the safety profile, optimal dosing strategies, and therapeutic efficacy of hesperidin in patients with MASH and related metabolic disorders.

## Figures and Tables

**Table 1:** Pharmacokinetics of hesperidin based on public databases.

Oral Bioavailability	Drug Likeness	Caco-2 Permeability
13.33%	0.67	−2.03

**Table 2:** Predicted binding affinities of hesperidin to representative pathogenic targets and network-derived hub proteins.

Protein	PDB ID	Binding Energy (kcal/mol)
CD36	5LGD	−8.7
FGF21	5VAQ	−8.0
IL-6	5MJ3	−9.4
CCL2	3BKB	−8.4
PNPLA3	1R6J	−8.6
SREBF1	4KAK	−8.3
PPARR	5U46	−8.4
TGFB1	4X2G	−8.6
TNF	7OUB	−9.2
AIFM1	4LII	−10.2
GSR	1Q1R	−10.0
BAX	9FI9	−8.8
AKT1	4GV1	−8.7
HMOX1	4G7L	−8.6
CDKN1B	6ATH	−8.4
ICAM1	2OZ4	−10.0
PPARGC1A	8BF1	−8.6
ALB	7VR0	−9.6
NOS3	3N5W	−8.8
CASP3	2CJY	−8.5
NOS2	5XN3	−7.1
PTGS2	3NT1	−11.2
JUN	4X21	−8.6

For targets with multiple PDB structures evaluated, the structure demonstrating the strongest binding affinity is presented. Binding energies are expressed in kcal/mol, with more negative values indicating stronger predicted interactions.

**Table 3:** Effects of hesperidin on body weight, daily food intake, and caloric intake in MCD-fed mice.

	NC	MCD	HES 150	HES 300	PIO
Body Weight (g)					
Baseline	25.00 ± 0.32	25.00 ± 0.33	25.40 ± 0.43	24.94 ± 0.48	25.30 ± 0.39
Week 1	26.73 ± 0.70	22.69 ± 0.37	23.64 ± 0.67	22.56 ± 0.52	22.72 ± 0.23
Week 2	28.43 ± 0.68	21.42 ± 0.36	21.94 ± 0.72	21.26 ± 0.67	21.02 ± 0.28
Week 3	30.03 ± 0.58	20.62 ± 0.37	20.90 ± 0.67	20.56 ± 0.78	19.85 ± 0.55
Week 4	30.35 ± 0.64	20.01 ± 0.26	20.28 ± 0.80	19.88 ± 0.95	19.30 ± 0.82
Daily oral intake (g)	2.90 ± 0.15	4.01 ± 0.20 ***	3.96 ± 0.27	4.59 ± 0.35 ***	4.49 ± 0.13 **
Daily calorie intake (kcal)	8.45 ± 0.43	16.73 ± 0.85 ***	16.55 ± 1.06	19.16 ± 1.47 ***	18.74 ± 0.52 **
Epididymal Fat Pads Weight (g)	0.42 ± 0.04	0.22 ± 0.02 ***	0.32 ± 0.09 *	0.39 ± 0.08 ***	0.22 ± 0.10
Fat/BW	1.37 ± 0.09	1.11 ± 0.09	1.64 ± 0.52 *	1.94 ± 0.36 ***	1.11 ± 0.45
Liver weight	1.50 ± 0.12	0.90 ± 0.02 ***	1.12 ± 0.09 **	1.14 ± 0.12 ***	0.73 ± 0.10 **
Liver/BW	4.93 ± 0.39	4.54 ± 0.11	5.46 ± 0.52 ***	5.67 ± 0.38 ***	3.78 ± 0.39 **

Data are expressed as the mean ± SEM (*n* = 5–10 per group). Statistical significance is indicated as * *p* < 0.05, ** *p* < 0.01, *** *p* < 0.001 vs. MCD group. NC, normal chow; MCD, methionine- and choline-deficient diet; HES 150/300, MCD + hesperidin 150 or 300 mg/kg/day; PIO, MCD + pioglitazone 30 mg/kg/day.

**Table 4:** Effects of hesperidin on serum hepatic injury biomarkers and lipid profiles.

	NC	MCD	HES 150	HES 300	PIO
AST (IU/L)	72.25 ± 3.94	557.56 ± 28.10 ***	391.2 ± 69.78 ***	369.2 ± 54.57 ***	243.5 ± 13.88 ***
ALT (IU/L)	28.25 ± 1.11	324 ± 25.81 ***	188.4 ± 49.11 ***	152.8 ± 37.78 ***	115.25 ± 12.62 ***
Creatinine (mg/dL)	0.23 ± 0.01	0.42 ± 0.02 ***	0.39 ± 0.04	0.416 ± 0.04	0.33 ± 0.03 ***
TC (mg/dL)	135.5 ± 5.52	42.33 ± 3.44 ***	45.4 ± 11.14	46.8 ± 13.51 ***	42.2 ± 5.15
HDL-C (mg/dL)	103.13 ± 6.08	28.93 ± 2.95 ***	33.28 ± 8.87	32.94 ± 10.88	28.08 ± 3.37
LDL-C (mg/dL)	13.75 ± 1.38	6.11 ± 0.59 ***	6.2 ± 1.11	6.2 ± 1.96	8.4 ± 1.25
TG (mg/dL)	184.75 ± 25.79	93.89 ± 4.17 ***	87 ± 11.26	83.4 ± 5.57	57.4 ± 3.12 ***
PL (mg/dL)	280.75 ± 10.14	114.33 ± 7.92 ***	120.4 ± 21.81	128.6 ± 22.23	98 ± 10.53 *
FFA (μmol/L)	3246.5 ± 272.38	1071.67 ± 51.26 ***	1027.4 ± 189.93	1109 ± 153.43	813.2 ± 50.95 *

Data are expressed as the mean ± SEM (*n* = 5–10 per group). Statistical significance is indicated as * *p* < 0.05, *** *p* < 0.001 vs. MCD group. AST, aspartate aminotransferase; ALT, alanine aminotransferase; TC, total cholesterol; TG, triglycerides; HDL-C, high-density lipoprotein cholesterol; LDL-C, low-density lipoprotein cholesterol; PL, phospholipids; FFA, free fatty acids. Reference ranges for C57BL/6 mice were as follows: AST, 50–298 IU/L; ALT, 20–60 IU/L; Creatinine, 0.1–0.5 mg/dL [27]; TC, 70–150 mg/dL; HDL-C, 50–80 mg/dL [28]; LDL-C, 19 mg/dL TG, 40–170 mg/dL; PL, 200–260 mg/dL [29]; FFA, 100–600 μmol/L [30].

**Table 5:** Regulatory effects of hesperidin on hepatic mRNA expressions involved in lipogenesis, inflammation, fibrosis, and oxidative stress.

	NC	MCD	HES 150	HES 300	PIO
*Srebf1*	1.01 ± 0.01	0.47 ± 0.03 ***	0.38 ± 0.02 **	0.62 ± 0.07 ***	0.45 ± 0.07
*Fasn*	1.01 ± 0.04	0.32 ± 0.03 ***	0.41 ± 0.15	0.27 ± 0.03	0.32 ± 0.05
*Ppara*	1.01 ± 0.01	0.64 ± 0.03 ***	0.58 ± 0.06 *	0.48 ± 0.02 ***	0.52 ± 0.06 ***
*Cpt1a*	1.02 ± 0.02	0.59 ± 0.06 ***	0.47 ± 0.05 **	0.52 ± 0.04	0.53 ± 0.06
*Adgre1*	1.00 ± 0.00	9.12 ± 0.44 ***	8.59 ± 0.88	6.89 ± 0.75 ***	6.77 ± 0.36 ***
*Ccl2*	1.02 ± 0.02	9.02 ± 0.49 ***	7.81 ± 1.01 *	6.00 ± 0.75 ***	5.50 ± 0.90 ***
*Tnf*	1.02 ± 0.02	7.90 ± 0.93 ***	6.04 ± 0.51 **	4.74 ± 0.51 ***	4.42 ± 1.08 ***
*Il6*	1.00 ± 0.00	1.28 ± 0.16 **	1.00 ± 0.00 **	1.10 ± 0.13	1.17 ± 0.13
*Il10*	1.03 ± 0.03	1.15 ± 0.12	1.01 ± 0.02	1.17 ± 0.13	1.23 ± 0.14
*Col3a1*	1.00 ± 0.00	2.56 ± 0.26 ***	2.00 ± 0.44 *	2.07 ± 0.13	2.27 ± 0.46
*Acta2*	1.00 ± 0.01	2.13 ± 0.20 ***	1.80 ± 0.37	1.93 ± 0.11	1.82 ± 0.23
*Tgfb1*	1.01 ± 0.01	2.00 ± 0.31 ***	1.40 ± 0.24 **	1.36 ± 0.23 ***	1.50 ± 0.20 **
*Timp1*	1.00 ± 0.00	11.84 ± 0.63 ***	8.81 ± 1.20 ***	8.17 ± 1.14 ***	8.48 ± 1.09 ***
*Sod2*	0.99 ± 0.01	0.62 ± 0.05 ***	0.58 ± 0.11	0.80 ± 0.06 **	0.58 ± 0.13
*Cat*	1.01 ± 0.01	0.56 ± 0.07 ***	0.51 ± 0.03	0.65 ± 0.07 *	0.49 ± 0.04
*Cyp2e1*	1.01 ± 0.01	3.62 ± 0.37 ***	2.22 ± 0.57 ***	2.14 ± 0.27 ***	2.82 ± 0.22 **

Relative hepatic gene expression levels were quantified by qRT-PCR, normalized to GAPDH, and calculated using the 2^−ΔΔCt^ method. Data are expressed as the mean ± SEM (*n* = 5–10 per group). Statistical significance is indicated as * *p* < 0.05, ** *p* < 0.01, *** *p* < 0.001 vs. MCD group. NC, normal chow; MCD, methionine- and choline-deficient diet; HES 150/300, MCD + hesperidin 150 or 300 mg/kg/day; PIO, MCD + pioglitazone 30 mg/kg/day. *Srebf1*, sterol regulatory element-binding transcription factor 1; *Fasn*, fatty acid synthase; *Ppara*, peroxisome proliferator-activated receptor alpha; *Cpt1a*, carnitine palmitoyltransferase 1A; *Adgre1*, adhesion G protein-coupled receptor E1; *Ccl2*, C-C motif chemokine ligand 2; *Tnf*, tumor necrosis factor; *Il6*, interleukin 6; *Il10*, interleukin 10; *Col3a1*, collagen type III alpha 1 chain; *Acta2*, actin alpha 2, smooth muscle; *Tgfb1*, transforming growth factor beta 1; *Timp1*, TIMP metallopeptidase inhibitor 1; *Sod2*, superoxide dismutase 2; *Cat*, catalase; *Cyp2e1*, cytochrome P450 family 2 subfamily E member 1.

## Data Availability

The original contributions presented in this study are included in the article. Further inquiries can be directed to the corresponding author.

## Abbreviations

The following abbreviations are used in this manuscript:

AEC	3-amino-9-ethylcarbazole
ALT	Alanine aminotransferase
ANOVA	Analysis of variance
AST	Aspartate aminotransferase
DAB	3,3′-diaminobenzidine
DL	Drug-likeness
FACS	Fluorescence-activated cell sorting
FFA	Free fatty acids
GAPDH	Glyceraldehyde-3-phosphate dehydrogenase
GFAP	Glial fibrillary acidic protein
GO	Gene Ontology
H&E	Hematoxylin and eosin
HDL-C	High-density lipoprotein cholesterol
HES	Hesperidin
IBA-1	Ionized calcium-binding adaptor molecule 1
IHC	Immunohistochemistry
KEGG	Kyoto Encyclopedia of Genes and Genomes
LDL-C	Low-density lipoprotein cholesterol
MASH	Metabolic dysfunction-associated steatohepatitis
MASLD	Metabolic dysfunction-associated steatotic liver disease
MCD	Methionine- and choline-deficient
NC	Normal chow
NEFA	Non-esterified fatty acids
OB	Oral bioavailability
PIO	Pioglitazone
PL	Phospholipids
PPI	Protein–protein interaction
qRT-PCR	Quantitative real-time polymerase chain reaction
SEM	Standard error of the mean
SMILES	Simplified molecular-input line-entry system
TC	Total cholesterol
TCMSP	Traditional Chinese Medicine Systems Pharmacology
TG	Triglycerides
VLDL	Very-low-density lipoprotein
